# Ovarian Teratoma Presenting With Gliomatosis Peritonei and a Melange of Symptoms Mimicking Ovarian Cancer in a Paediatric Patient

**DOI:** 10.7759/cureus.49945

**Published:** 2023-12-05

**Authors:** Vishal Bahall, Lance De Barry, Rachael Sookdeo, Mickhaiel Barrow

**Affiliations:** 1 Obstetrics and Gynaecology, The University of the West Indies, Saint Augustine, TTO; 2 Obstetrics and Gynaecology, San Fernando General Hospital, San Fernando, TTO; 3 Pathology, Port of Spain General Hospital, Port of Spain, TTO

**Keywords:** surgery, pediatrics, gynecology, gliomatosis peritonei, ovarian teratoma

## Abstract

Gliomatosis peritonei (GP) is a rare condition characterised by mature glial nodules that implant in the peritoneum, lymph nodes, or omentum. GP is typically associated with mature or immature ovarian teratomas and usually affects adolescent females. Although neuroglia may be a standard feature of mature ovarian teratomas, widespread peritoneal glial nodules, ascites, and pleural effusion are rare, particularly in the paediatric population.

We report a case of a giant left mature ovarian teratoma associated with GP and omental splenunculus in a 12-year-old female who presented with constipation, an adnexal mass, ascites, pleural effusion, and elevated CA-125 levels. The patient successfully underwent fertility-sparing surgery in the form of a left salpingo-oophorectomy, omentectomy, and resection of peritoneal glial deposits. In light of the current scarcity of data on this clinical entity in the literature, we hope to raise awareness of this rare presentation of mature ovarian teratoma, the challenges associated with preoperative diagnosis, and the impact of fertility-sparing surgery on potential oncological and reproductive outcomes in a paediatric patient.

## Introduction

Ovarian teratomas are the most common type of ovarian mass that occurs in childhood and adolescence, accounting for 50% of paediatric tumours [1]. Ovarian teratomas are embryonal neoplasms with two or more germ layers present [1]. According to WHO, ovarian teratomas may be classified as mature or immature, with the latter carrying a potential for malignant transformation [2]. Mature cystic ovarian teratomas (MCOT) or dermoid cysts account for 70% of benign ovarian tumours in women below 30 years of age [1]. While the clinical symptoms of mature ovarian teratomas vary widely, 20% of tumours are asymptomatic and may be detected incidentally during pelvic imaging [3]. Large tumours may present with abdominal pain, symptoms of increased pelvic pressure, a palpable mass on examination, nausea, vomiting, bloating, and torsion [3]. Gliomatosis peritonei (GP) is a rare condition associated with ovarian teratoma [4]. Patients with MCOT accompanied by GP may manifest a rare clinical picture that resembles pseudo-Meigs syndrome - a condition marked by ovarian teratoma, ascites, and pleural effusion [5].

Due to its rarity, MCOT with GP poses a diagnostic challenge as it may easily be mistaken for a malignant ovarian mass such as a germ cell tumour, particularly in the paediatric population [6]. We report a rare case of a giant mature ovarian teratoma accompanied by GP, massive ascites, pleural effusion, and splenunculus in a 12-year-old female patient. The patient’s constellation of signs and symptoms presented a significant diagnostic challenge, due to its resemblance to a malignant adnexal mass on preoperative assessment, and a therapeutic dilemma regarding the impact of choice of surgery on potential oncological and reproductive outcomes. Nevertheless, while fertility-sparing surgery may cause concerns for poor oncological outcomes, radical surgery can negatively impact future fertility and hence a multidisciplinary approach to treatment is necessary.

## Case presentation

A 12-year-old female presented to the Gynaecology department, accompanied by her mother, with complaints of chronic constipation and evolving abdominal distension for three months. She reported occasional nausea and vomiting with bloating, vague abdominal pain, and generalised malaise. The patient denied weight loss, decreased appetite, vaginal bleeding, or urinary symptoms. She had no medical, gynaecological, or surgical history, had begun regular menses one year earlier, and had met all expected developmental milestones. The patient had no personal or familial history of malignancy.

On clinical examination, the abdomen was tense and distended, and a mass arising from the left aspect of the pelvis was noted. Laboratory investigations were within normal limits, including a complete blood count and renal and liver function tests. The tumour marker - cancer antigen CA-125 - was elevated (237 IU), while other tumour markers, including carcinoembryonic antigen (CEA), CA-19-9, and alpha-fetoprotein (AFP) were all normal within normal limits. Pelvic ultrasonography highlighted a left complex adnexal mass measuring approximately 18 x 20 cm with mixed cystic and solid components and gross ascites. There were no signs of left hydroureter, hydronephrosis, or pelvic lymphadenopathy.

A CT scan of the chest, abdomen, and pelvis was performed to characterise the adnexal mass and plan further treatment. Abdominopelvic CT demonstrated a large volume of free fluid distending the abdominal cavity with a widespread omental and peritoneal nodularity suggestive of metastatic lesions. A large fluid-filled mass measuring 21 x 18 x 11 cm was highlighted with thin septated walls and variable calcifications - likely an immature teratoma. The uterus measured 4 x 3.4 cm, and the contralateral adnexae appeared unremarkable. There was no evidence of pelvic lymphadenopathy. Chest CT demonstrated bilateral pleural effusion, more pronounced in the left hemithorax, with trace pericardial effusion. These findings suggested a malignant ovarian tumour - likely a germ cell or ovarian teratoma. Treatment options were discussed with the patient and her parents, including the impact of surgery on potential oncological outcomes and future reproductive potential. The patient assented, and her parents consented to a left salpingo-oophorectomy, omentectomy, resection of peritoneal nodules, and peritoneal washings.

**Figure 1:**
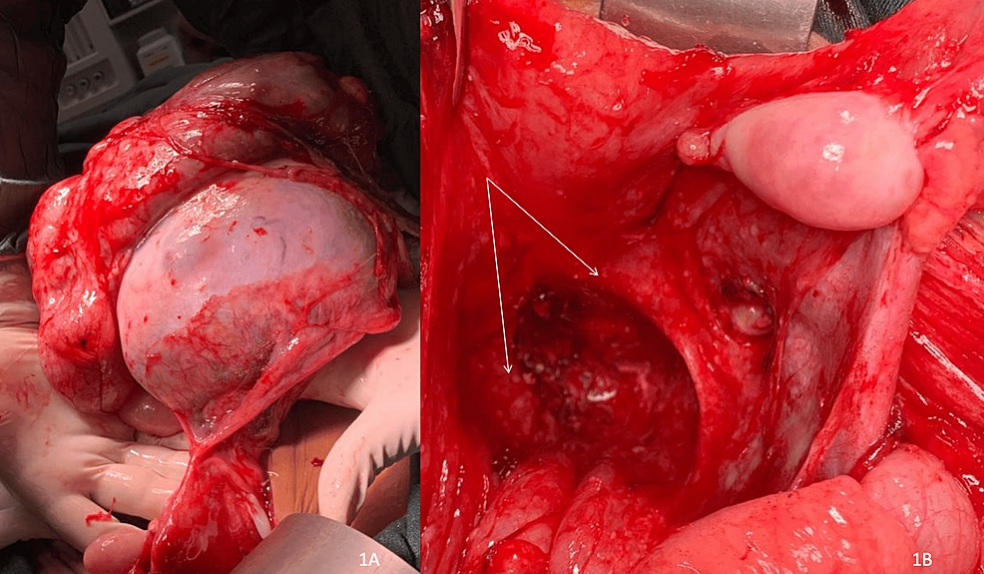
(1A) Giant left mature cystic ovarian teratoma adherent to omentum. (1B) Mature glial peritoneal deposits in the pouch of Douglas and uterosacral ligaments (white arrows)

Intraoperatively, the abdomen was opened by a supraumbilical midline incision after a rectus sheath block was performed. Approximately 3.5 L of straw-coloured ascitic fluid was evacuated, and a sample was sent for peritoneal cytology. The left adnexal mass observed on presurgical imaging was multilocular, contained cystic and solid components, measured approximately 20 x 18 cm and was densely adherent to the omentum (Figure 1A). Numerous nodules of varying sizes were noted on the peritoneum and omentum (Figure 1B). The right fallopian tube and ovary appeared unremarkable. A left salpingo-oophorectomy and omentectomy were performed. Additionally, peritoneal nodules were excised, particularly from the pouch of Douglas, and sent for histological examination. An abdominal drain was inserted, and the estimated intraoperative blood loss was less than 100 ml.

**Figure 2:**
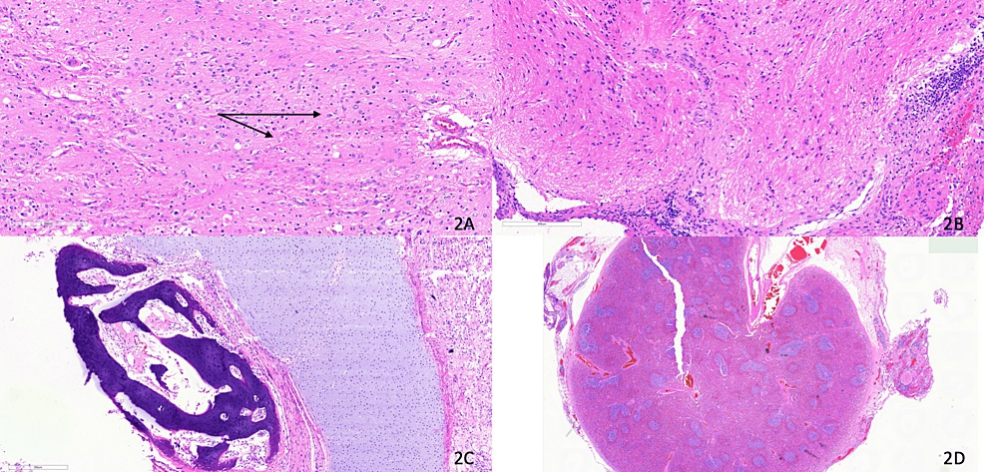
Histopathology (2A) Mature cystic ovarian teratoma comprising cartilage, skin, fat, and glial tissue (x200). (2B) Nodule of mature glial tissue in the pouch of Douglas (gliomatosis peritonei) (x200). (2C) Section of cartilage and bone contained within mature cystic ovarian teratoma (x100). (2D) Section of ectopic spleen embedded in the omentum (x10)

Histopathology of the left adnexal mass demonstrated a benign mature teratoma that included components of skin, adipose tissue, glial tissue, respiratory and glandular epithelium, bone, and cartilage (Figures 2A, 2C). There was no evidence of immature elements or somatic malignancy, and sections from the fallopian tube were unremarkable. The omental specimen demonstrated accessory splenic tissue (splenunculus) with no evidence of malignancy and several benign lymph nodes (Figure 2D). At the same time, the peritoneal nodules contained mature glial tissue surrounded by fibrous tissue and chronic inflammation - findings indicative of GP (Figure 2B). Peritoneal cytology highlighted epithelial cells on a background of lymphoid cells and histiocytes. These findings confirmed the diagnosis of mature ovarian teratoma accompanied by GP and omental splenunculus.

The patient's postoperative course was unremarkable, and she was discharged in satisfactory condition one day later. Although she was admitted again on the fourth postoperative day for paralytic ileus, her symptoms improved, and she was discharged again in satisfactory condition on the fifth day. The abdominal drain produced a daily output of 50-60 ml of straw-coloured ascitic fluid for approximately one week after surgery, and it was subsequently removed on the 10th day. At her postoperative follow-up visits, the patient was well and had no recurrence of her symptoms or ascites on ultrasound.

## Discussion

GP is an exceedingly rare disease characterised by peritoneal or omental implantation of mature glial tissue [4]. While GP is predominantly associated with immature ovarian teratomas, there have been documented cases of GP occurring in tandem with mature ovarian teratomas [4]. It was first described by Neuhauser in 1905, and fewer than 100 cases have been described in the literature in English since then [3]. It is a benign condition with rudimentary potential for malignant transformation [4]. MCOT accompanied by GP primarily affects adolescent females aged 11-22 years [7]. According to a case series of MCOT associated with GP by Yoon et al., the median age at diagnosis was 13 years [7]. The overall prevalence of MCOT associated with GP is unknown, mainly because this condition is often underdiagnosed.

The aetiological mechanisms of GP occurring in tandem with MCOT have yet to be well understood; however, two theories have been proposed to explain the possible presence of glial peritoneal and omental implants. The first theory relates to capsular defects of the primary ovarian teratoma or dissemination of glial tissue through angiolymphatic vessels [8]. Direct seeding of glial tissue may explain GP within the omentum, particularly in cases where the omentum was adherent to the teratoma [8]. In an analysis by Robboy et al., this mechanism may have accounted for 11 out of 12 cases of glial deposits within the omentum, either from capsular defects of the teratoma or adherence of the omentum to the surface of the teratoma, which was likely the aetiopathogenesis in our case [8]. Another theory suggests that glial foci are genetically unassociated with ovarian teratomas [9]. It may be plausible that peritoneal stem cells may differentiate into glial cells under direct stimulation of certain neuroendocrine factors secreted by teratomas [9]. However, the detailed mechanism by which peritoneal cells develop into glial follicles remains largely undetermined.

Most cases of MCOT associated with GP are asymptomatic and may be found incidentally on pelvic imaging. MCOT may present as a slowly enlarging adnexal mass associated with pelvic pain or pressure, nausea, vomiting, constipation, bloating, or decreased appetite [10]. In some cases, MCOT may undergo torsion. The presence of massive ascites or pleural or pericardial effusions represents a rare association of MCOT with GP [5]. The clinical presentation of massive ascites, pleural effusion, and an ovarian mass in a young female may raise concerns for Meigs syndrome or ovarian cancer [5].

MCOT with GP poses a unique diagnostic challenge as it resembles more sinister conditions like ovarian malignancy, germ cell tumour, metastatic cancer, Meigs syndrome, miliary tuberculosis, carcinomatosis, or even endometriosis [11]. Although surgical resection with histological assessment provides a definitive diagnosis, MCOT with GP may be suspected based on a combination of radiologic, biochemical, and cytological analyses. CT or MRI may highlight a complex, septated adnexal mass, peritoneal or omental nodules, ascites, and pleural or pericardial effusions [6]. A paracentesis specimen may appear straw-coloured and demonstrate the presence of epithelial cells, lymphoid cells, and histiocytes, and the absence of malignant cells. In some cases, the serum cancer antigen CA-125 may be elevated - a non-specific finding associated with a range of gynaecologic and non-gynaecologic conditions.

Histologically, GP is diagnosed on haematoxylin and eosin-stained tissue sections that demonstrate mature glial tissue often surrounded by fibrous tissue [10]. A positive immunohistochemical staining reaction of glial tissue for the neural marker glial fibrillary acidic protein (GFAP) may be utilised as an adjunct with an intense expression suggesting mature, well-differentiated tumour cells [11]. MCOTs are typically lined by squamous epithelium with a cystic component predominantly filled with sebaceous material. On gross appearance, glial implants are generally 1-10 mm in size, while mature cystic tumours have no characteristic shape or size; however, hair, teeth, or bone may be identifiable on examination [11]. Microscopic examination may confirm the presence of tissue derived from two or more germ cell layers, including epithelium and neural tissue, muscle, fat, bone or cartilage, thyroid tissue, and gastrointestinal epithelium [1].

MCOT with GP is a benign condition, and treatment beyond what is appropriate for the parent ovarian teratoma has not been recommended. The impact of surgical resection and its influence on oncological and reproductive outcomes is one source of contention, particularly when planning treatment for paediatric patients. Fertility-sparing surgery is the standard surgical approach and involves salpingo-oophorectomy, omentectomy, and multiple biopsies of the peritoneal deposits [12]. In most cases, GP is extensive and complete resection is technically challenging [12]. According to Bentivegna et al., residual mature GP deposits are asymptomatic and may be inert for a long period of time [13]. However, if immature glial tissue is present, the treatment algorithm for metastatic ovarian teratoma should be implemented [13]. MCOT with GP is associated with a favourable prognosis mainly if the histological nature of glial deposits is entirely mature, or if there is a loss of proliferative activity of the peritoneal implants [14]. However, long-term follow-up is recommended since malignant transformation of glial peritoneal deposits has been documented [14]. According to Lavoie et al., fluorodeoxyglucose (FDG) positron emission tomography (PET)/CT may be useful for the follow-up of GP [15]. There are currently no standardised guidelines on the duration and method of follow-up.

## Conclusions

GP associated with MCOT is an exceedingly rare condition, particularly in paediatric patients. Moreover, the presence of ascites and pleural or pericardial effusion constitutes a highly unusual presentation of this clinical entity. This case posed a diagnostic challenge as the constellation of an adnexal mass, widespread peritoneal deposits, ascites, and pleural effusion led to a suspicion of advanced ovarian malignancy in a paediatric patient. Histopathologists play a critical role in the confirmation of MCOT with GP, especially because there is an overall low suspicion of GP on the part of clinicians. While standardised protocols are unavailable for the treatment and follow-up of MCOT with GP, a fertility-sparing surgical approach with long-term follow-up appears to confer the best patient outcomes, especially in cases where the GP deposits are mature.
